# Assessment of Imputation from Low-Pass Sequencing to Predict Merit of Beef Steers

**DOI:** 10.3390/genes11111312

**Published:** 2020-11-05

**Authors:** Warren M. Snelling, Jesse L. Hoff, Jeremiah H. Li, Larry A. Kuehn, Brittney N. Keel, Amanda K. Lindholm-Perry, Joseph K. Pickrell

**Affiliations:** 1 U.S. Department of Agriculture, Agricultural Research Service, U.S. Meat Animal Research Center, Clay Center, NE 68933, USA; larry.kuehn@usda.gov (L.A.K.); brittney.keel@usda.gov (B.N.K.); amanda.lindholm@usda.gov (A.K.L.-P.); 2 Gencove, Inc., New York, NY 10016, USA; jesse.hoff@gencove.com (J.L.H.); Jeremy.li@gencove.com (J.H.L.); joseph.pickrell@gencove.com (J.K.P.)

**Keywords:** sequence, imputation, genomic prediction, beef cattle

## Abstract

Decreasing costs are making low coverage sequencing with imputation to a comprehensive reference panel an attractive alternative to obtain functional variant genotypes that can increase the accuracy of genomic prediction. To assess the potential of low-pass sequencing, genomic sequence of 77 steers sequenced to >10X coverage was downsampled to 1X and imputed to a reference of 946 cattle representing multiple *Bos taurus* and *Bos indicus*-influenced breeds. Genotypes for nearly 60 million variants detected in the reference were imputed from the downsampled sequence. The imputed genotypes strongly agreed with the SNP array genotypes ($\bar{r}=0.99$) and the genotypes called from the transcript sequence ($\bar{r}=0.97$). Effects of BovineSNP50 and GGP-F250 variants on birth weight, postweaning gain, and marbling were solved without the steers’ phenotypes and genotypes, then applied to their genotypes, to predict the molecular breeding values (MBV). The steers’ MBV were similar when using imputed and array genotypes. Replacing array variants with functional sequence variants might allow more robust MBV. Imputation from low coverage sequence offers a viable, low-cost approach to obtain functional variant genotypes that could improve genomic prediction.

## 1. Introduction

Current genomic evaluations of beef cattle use genotypes from commercial SNP arrays to predict breeding values with greater accuracy than breeding values predicted using only pedigree and performance records. Further increases in accuracy, particularly for multi-breed populations, can be achieved by including functional sequence variants [1,2,3]. Obtaining the functional variant genotypes needed to increase accuracy, however, is a challenge. One array to genotype potentially functional variants is available [4], but it is missing much of the functional variation detected in the sequence of beef cattle [5], and many alleles probed by that array are too rare to be informative. One intent of sequencing efforts is to provide a reference for imputation from array genotypes to sequence variants, but the disparity in allele frequency distributions of array and sequence variants [4,6] limits imputation accuracy, especially for the rare variants. Low-pass (<1X) sequence is not subject to the same limitation and is imputed to comprehensive sets of sequence variants with high accuracy [7,8]. Decreasing sequencing costs [9] coupled with highly multiplexed library preparation methods [10] make low-pass sequencing (LPS) cost-competitive with SNP arrays, and provides a straightforward approach to impute functional variant genotypes, without complications of variant selection, probe design, and call training associated with developing SNP arrays [11]. This study was conducted to evaluate the potential of LPS in beef cattle, using existing sequence data to mimic LPS before submitting a large number of samples through an LPS and imputation pipeline.

## 2. Materials and Methods 

### 2.1. Data Source

Data for this study were obtained from the on-going U.S Meat Animal Research Center (USMARC) Germplasm Evaluation Project (GPE). Animals were raised, and biological samples for genotyping and sequencing were obtained following the USMARC standard operation procedures and Federation of Animal Science Societies (FASS) guidelines [12]. Pedigree (*n* = 120,207), birth weight (*n* = 78,625), postweaning gain (*n* = 68,846), and marbling score (*n* = 38,850) records from GPE animals were extracted from the USMARC cattle records database. A 19,420-animal subset of the GPE population was genotyped with at least one genotyping array (Table 1). Whole-genome sequence (WGS) was available from 77 of the 80 GPE steers selected for transcriptome sequencing from individual feed intake and gain records [13]. 

Data included records from the eight historic cycles of GPE and the on-going continuous GPE project. Starting in 1968, the cycles were breed for comparison experiments, with the base cows artificially inseminated (AI) to industry sires, representing five to seven breeds. Each cycle included Angus and Hereford industry sires, and USMARC Angus and Hereford base cows; MARC III composite cows [14] were introduced in later cycles. Cycle VII was a re-evaluation of the seven breeds (Angus, Charolais, Gelbvieh, Hereford, Limousin, Red Angus, and Simmental) that were the most influential in the U.S. beef industry [15], and transitioned into the current continuous GPE project [16]. Sires from 18 breeds were periodically sampled, and the female progeny mated to their breed-of-sire to produce breeding females that are a high percentage (>87.5%) of one of the 18 breeds. The 18 breeds included the Cycle VII breeds, and 11 others that conduct national cattle evaluations (NCE) for beef production traits (Beefmaster, Brahman, Brangus, Braunvieh, ChiAngus, Maine-Anjou, Salers, Santa Gertrudis, Shorthorn, South Devon, and Tarentaise). 

According to the recorded pedigree, the 77 steers with WGS had contributions from 20 different breeds, and were sired by 70 different registered bulls representing 17 breeds (all continuous GPE breeds except Tarentaise). Other breeds contributing to the steers included Pinzgauer, Red Poll, and Holstein. Eighteen steers with MARC III ancestors had up to 7% Pinzgauer and Red Poll, and one was 2% Holstein, tracing to a twinning study at USMARC [17]. Six steers were purebred, three Angus, and three Hereford. Twenty were crosses of the predominant Cycle VII breeds, 26 had contributions from other *Bos taurus* breeds, and 25 had *Bos indicus* influence from Brahman or one of the *indicus*-influenced composites, Beefmaster, Brangus, or Santa Gertrudis. Sixteen steers were sired by one of the 14 sequenced bulls included in the cattle haplotype reference (Table S1).

### 2.2. SNP Array Genotypes

Genotyped animals represented Cycle VII and continuous GPE. Most sires (AI and natural service) were genotyped with both the BovineHD (HD; ~770K SNP) and GGP-F250 (F250; ~200K SNP including ~170K putative functional variant) assays. Additional animals genotyped with those assays included dams whose sires were not genotyped, and some non-parents with phenotypes for difficult-to-measure traits. Other dams were genotyped with the BovineSNP50 (50K), and non-parents with lower density GGP assays. Genotyped DNA was extracted from AI sires’ semen, blood from USMARC-born single-birth animals, and an ear notch from recorded twins. The sequenced steers were genotyped with different arrays, 41 with the 50 K, 28 with a lower density GGP, 6 with the F250 and HD arrays, and 2 with the F250 and a GGP array.

Prior to pedigree imputation with findhap version 3 [18], genotypes were filtered for call rate (>0.95 by animal and variant) and minor allele frequency (>0.005). Variants were ordered by position on the ARS-UCD1.2 genome assembly [19], using the coordinates provided in the National Animal Genome Research Program (NAGRP) Community Data Repository [20]. All animals with genotypes from any assay were imputed to the combined BovineHD and GGP-F250 variant set. Functional variant genotypes of 300 2013-born nonparents with F250 genotypes were discarded from the first round of imputation, as a test of imputation accuracy. Functional variants with correlations <0.95 between the imputed and assayed genotypes of the 300 test animals were removed for the final round of imputation. The final round imputed genotypes of the 748,804 variants located on autosomes and the pseudoautosomal region of the X chromosome (paX) for 18,327 animals. 

### 2.3. Genetic Prediction

Breeding values were predicted for birth weight (BW), postweaning average daily gain (PWG), and marbling score (MARB). The model for each trait was y=Xβ+Zu+e, with var(u) = ${A\sigma }_{g}^{2}$, var(e) = ${I\sigma }_{e}^{2}$, and cov(u, e) = 0; y is a vector of observations, X is an incidence matrix relating observations to the vector of fixed effects in β, Z is an incidence matrix relating observations to random additive genetic effects in u, and e is a vector of random residual effects; A is the numerator relationship matrix describing pedigree relationships among animals, I is an identity matrix, $\sigma_{g}^{2}$ is the variance of additive genetic effects, and $\sigma_{e}^{2}$ is the variance of residuals. Models to predict genomic breeding values were identical, except that A was replaced by a genomic relationship matrix G*. G was computed as ${G={\mathrm{MM}}^{\prime }/2\Sigma p}_{i}\left(1-p_{i}\right)$ [21], where M is a matrix of variant genotypes (0, 1, or 2 copies of allele B) and p_i_ is the B allele frequency for the ith variant. G* was scaled as 0.99G+0.01I to avoid singularity.

Birth weight fixed effects included the age of dam category (2 through 4.5 in half-year increments, 5 to 10 and > 10 years) and the contemporary group (CG) defined by calf sex, year, season, and location on the research center. Postweaning gain fixed effects included sex at weaning (distinguishing bulls from steers), and CG defined by year, season, and management group (ration, implant, weigh dates, etc.) from weaning through yearling (for females retained for breeding) or slaughter. The PWG observations were computed from all weights observed from weaning through 550 days of age. Following [22], a quadratic regression on age (days) was fitted for each individual, and weight 160 days post-weaning was projected to determine the average daily gain. The CG for MARB included PWG CG and slaughter date. Numeric MARB scores were assigned to USDA Degree of Marbling, with possible scores ranging from 0 (Devoid00) to 999 (Abundant99). 

Best linear unbiased prediction (BLUP) of breeding values for each trait assumed additive genetic (genomic) and residual variances, estimated with restricted maximum likelihood (REML) algorithms implemented in WOMBAT [23]. All animals, including the sequenced GPE steers, were predicted with pedigree relationships, and with the genomic relationships computed with all imputed genotypes. Phenotypes and genotypes of the sequenced steers were eliminated from the analyses to train variant effects, then to predict molecular breeding values (MBV) of the steers by applying the variant effects to their genotypes. For each trait, the effects were trained for three sets of variants—(1) variants probed by the 50K assay, (2) putative functional content of the F250, and (3) the most significant functional variants selected from 5000 permutations of F250-based breeding values [24]. Variant effects were solved by $\hat{\alpha }=M^{\prime }{\left[MM^{\prime }\right]}^{-1}\hat{u}$ [25], where $\hat{\alpha }$ is a vector of variant effects and $\hat{u}$ is a vector of additive genomic effects predicted with the G for a set of variants. For comparison to the breeding values predicted with pedigree and genomic relationships with all variants, steers’ MBV were then predicted by $\mathrm{MBV}=M_{s}\hat{\alpha }$, where Ms is a matrix of steers’ genotypes. The MBV were predicted with the genotypes obtained from the SNP arrays and the genotypes imputed from the downsampled WGS.

### 2.4. Low-Pass Sequence and Imputation

Ten million read pairs (~1X) per steer were randomly sampled from the >10X WGS available on the 77 sequenced GPE steers, using seqtk [26]. The downsampled sequence was submitted to the Gencove pipeline for imputation to the cattle haplotype reference panel with loimpute [27].

To build the reference panel for imputation of low-pass sequencing data, sequencing data from 946 animals from two sources were compiled (Table S1): publicly available sequence data available on the NCBI Short Read Archive, and sequenced samples in the GPE. These animals cover a range of dairy and beef cattle breeds. For each sample, FASTQ files were obtained and then:

1. Aligned the reads to the ARS-UCD1.2 genome using bwa mem v0.7.17 [28]
2. Sorted the reads using samtools v1.10 [29]
3. Marked duplicate reads using GATK version 4 [30] (MarkDuplicates)
4. Recalibrated base quality scores using GATK version 4 (BaseRecalibrator)
5. Called GVCF in 10Mb windows using GATK version 4 (HaplotypeCaller -ERC GVCF)

Variant calls were then generated and phased:

1. Called variants in the same 10Mb windows as above using the GATK version 4 (GenotypeGVCFs)
2. Filtered single nucleotide polymorphism calls using GATK version 4 (VariantFiltration) with the filter string ‘QD < 2.0 || FS > 60.0 || MQ < 40.0 || MQRankSum <−12.5 || ReadPosRankSum < −8.0′
3. Filtered indel calls using GATK version 4 (VariantFiltration) with the filter string ‘QD < 2.0 || FS > 200.0 || ReadPosRankSum < -20.0 || SOR > 10.0′
4. Refined variant calls using BEAGLE v4 [31]
5. Phased variant calls using BEAGLE v5 [32]
6. Filtered indels and multi-allelic sites.
7. Principal components were generated using plink 1.9 [33] restricted to 150,000 randomly-chosen bi-allelic SNPs with minor allele frequency (across the entire panel) above 5%.

Genotypes of SNP array variants were extracted from the variant call format (VCF) files written by the imputation pipeline. Identity of the sequencing libraries was confirmed by comparing imputed genotypes to array genotypes and the genotypes of the variants expressed in muscle transcriptome of each steer [34]. Additionally, a phred-scaled call confidence (CC) score was assigned to each steer as a measure of imputation quality. Genotype probabilities (GP) for each array variant listed in the VCF were extracted, and CC was computed as the mean 10 × log_10_(1−GP_max_) of each uncertain call (GP_max_ < 1), where GP_max_ is GP of the most probable of the three possible genotypes at a variant site. Functional impact of each variant was predicted with snpEff v4.3 [35], using ensemble annotation (release 96) of the ARS-UCD1.2 assembly [24]. Figure S1 depicts the general flow from the GPE project data and steer sequence through the MBV of the steers.

## 3. Results

### 3.1. Cattle Haplotype Reference Panel

**Figure 1:**
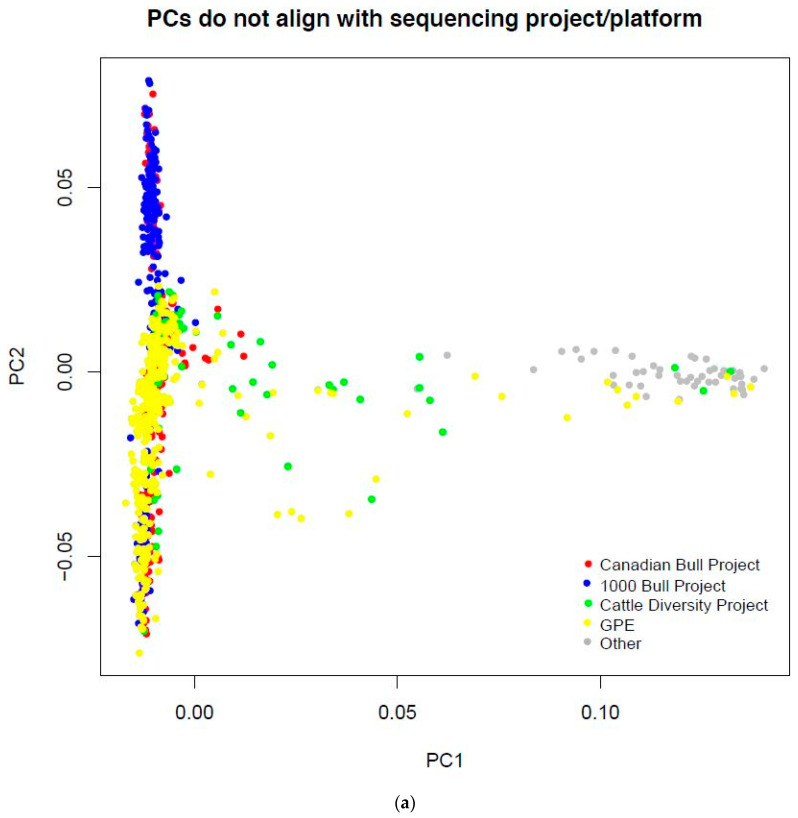

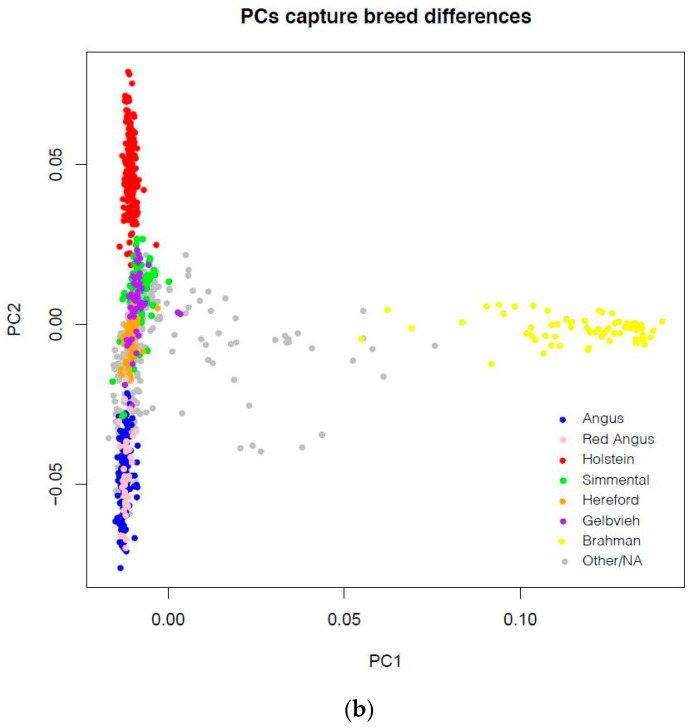
Principal component (PC) analysis of the haplotype reference panel. (**a**) Overlap among projects sequenced with different platforms; (**b**) depicts PC1 separating the *Bos indicus* from *Bos taurus* breeds, and PC2 separating Holstein from Angus, with other *Bos taurus* breeds intermediate between Holstein and Angus. The first two PC explained 11% of genomic relationships among the reference, 7% by PC1, and 4% by PC2.

Sequence contributing to the imputation reference was generated in different projects, using SOLiD and Illumina instruments. Principal component analysis (PCA) of the sequenced individuals showed considerable overlap among projects (Figure 1a), suggesting that sequence from the different projects and platforms could be combined to construct a haplotype reference panel. The main differences between projects where whether or not they included Holstein or *Bos indicus*-influenced animals (Figure 1b). The first principal component separated *Bos taurus* from *Bos indicus,* and indicated some variation in the individual separation of Brahman from *Bos taurus*. The second principal component separated Holstein from Angus, with other *Bos taurus* breeds intermediate between Holstein and Angus. Continental European breeds, such as Simmental and Gelbvieh, appeared closer to Holstein, and Hereford was closer to Angus. Various *Bos taurus* crossbreds in the reference were along the continuum between Continental breeds and Angus, and *Bos indicus* influenced crossbreds and composite breeds in the space between *Bos taurus* and Brahman.

### 3.2. Variants Imputed from Low-Pass Sequence

After filtering, 59,198,025 variants with a mean spacing of 44 bp were detected in the haplotype reference panel and imputed with the low-pass pipeline (Table S2). There were 332,714 variants, which were expected to alter the proteins coded by 21,066 of the 21,861 annotated protein-coding genes, and another 327,367 that might affect regulation of those genes (Table 2). Genotypes for 715,402 of the 748,804 usable autosomal and paX variants on the SNP arrays were imputed from the downsampled sequence.

On average, 98.9% of all genotypes called from the downsampled exceeded the GP_max_ > 0.9 threshold to pass imputation, and 92.6% of variants had pass rates greater than 95%. Low pass rates were most prevalent for BTA 23, which was the only chromosome with more than 10% of variants having pass rates less than 95%. Across the genome, every one-megabase (MB) interval contained variants with pass rates less than 95% (Table S3). More than 38% of the sites within the interval around BTA 23:26 MB, and within three consecutive intervals on BTA 10 around 23, 24, and 25 MB had pass rates less than 95%. The BTA 23:26 MB interval was the most variant-dense single MB interval of the genome, with the 77,545 variants separated by a mean of 12.9 bp between variants. This region contained part of the bovine major histocompatibility complex, containing highly polymorphic loci associated with immunity [36]. The BTA 10:23 and 10:25 MB intervals were relatively dense (18.3 to 24.3 bp mean separation) but with 58.5 bp between variants, the BTA 10:24 MB interval was less dense than the mean 47.3 ± 26.3 bp separation between variants.

**Figure 2:**
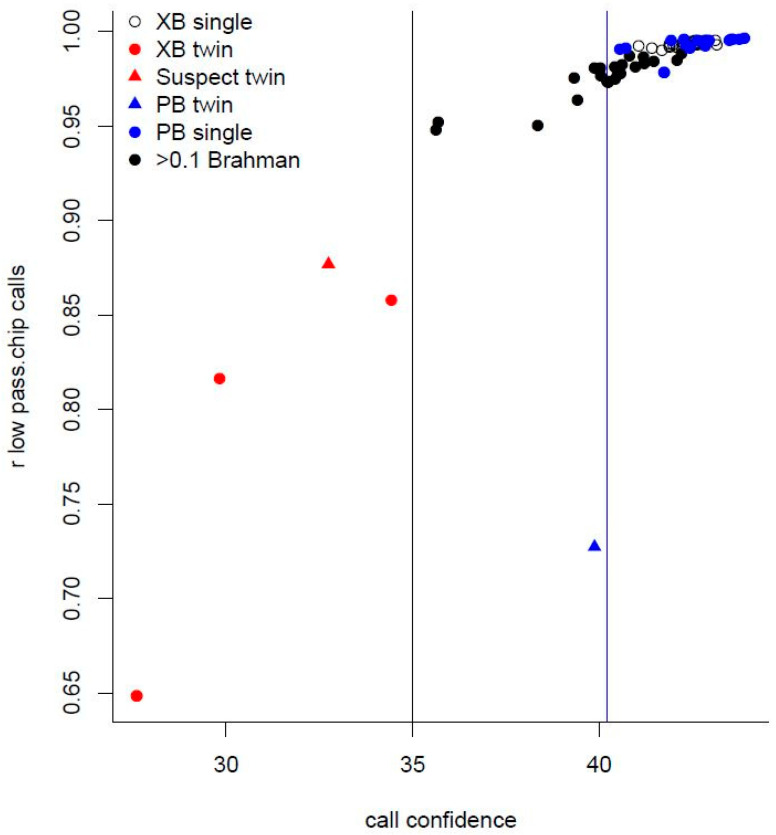
Relationship between imputation accuracy, expressed as a correlation (r) between genotypes imputed from sequence and called from SNP arrays, and call confidence—a function of imputed genotype probabilities. Accuracy and call confidence were lowest for the known crossbred (XB) steers, which were sequenced with DNA extracted from blood, another low-confidence, low-accuracy steer was suspected to be a twin. The purebred (PB) *Bos taurus* steer with lowest accuracy had the lowest call confidence of any *Bos taurus* and was a known twin. *Bos indicus*-influenced steers (>0.1 Brahman) tended to have lower call confidence and accuracy than *Bos taurus* steers.

None of the downsampled libraries had pass rates less than 95%. While the pass rate and CC scores rank libraries were similar (Spearman r = 0.90), the phred-scaled CC scores provided clearer separation between libraries. The CC scores were indicative of the agreement between the genotypes imputed from the downsampled sequence and called from SNP arrays. The libraries with noticeably lower CC also had a lower agreement between the sequence and array genotypes. Correlations between the sequence and array genotypes (r_sa_) were < 0.90 for libraries with CC < 36.6, and r_sa_ was > 0.95 for all but one library with CC > 37.6 (Figure 2).

**Figure 3:**
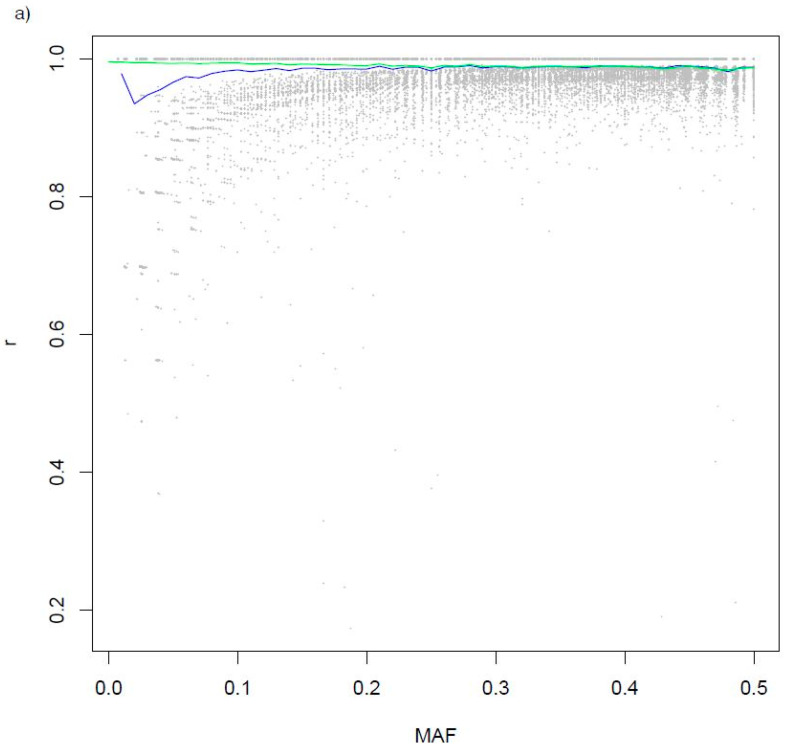

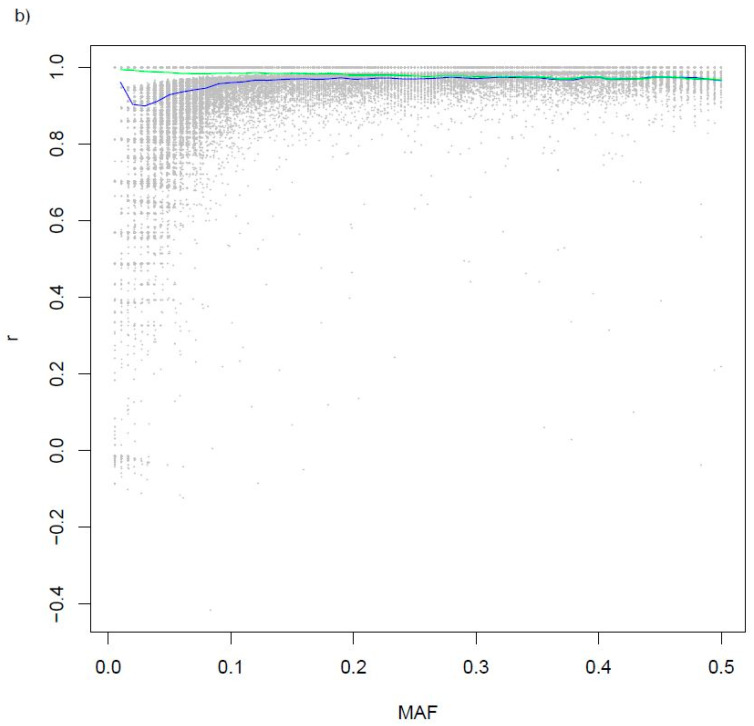
Relationship between imputation accuracy, expressed as a correlation (r) between genotypes imputed from sequence and called from SNP arrays (**a**) or transcript sequence (**b**), and minor allele frequency (MAF). Mean correlation between imputed and called genotypes within 0.01 MAF increments is shown by blue lines, and the green lines show mean concordance within the 0.01 MAF increments.

There was complete agreement between genotypes, which passed imputation from sequence and called from SNP arrays for 70% of the variants called for at least 35 steers (Figure 3a). The lowest mean r_sa_ within 0.01 minor allele frequency (MAF) increments was 0.93 at MAF = 0.02, and > 0.98 for all MAF increments > 0.08. Concordance between sequence and array calls was consistently > 0.98 for all MAF increments. Agreement between genotypes imputed from downsampled sequence and called from transcript sequence was somewhat less, but followed a similar pattern (Figure 3b). There was perfect agreement between the transcript and downsampled calls for about half the transcript variants. The lowest mean correlation between the downsampled sequence and transcript genotypes (r_st_) was in the MAF = 0.03 increment, with r_st_ = 0.90, and MAF increments > 0.08 had r_st_ > 0.95.

Call confidence and agreement between imputed sequence and array genotypes were strongly influenced by *Bos indicus*. Ignoring the steers with unusually low CC, *Bos indicus*-influenced steers had lower CC (*p* < 1e^−13^) and lower r_sa_ (*p* < 1e^−11^) than *Bos taurus* steers. Within the *Bos indicus*-influenced steers, when the pedigree contributions ranged from 12% to 85% Brahman, the amount of Brahman influence did not affect CC (*p* = 0.58) or r_sa_ (*p* = 0.10). Purebred steers and steers whose sire was in the haplotype reference had somewhat higher CC than crossbred steers (*p* = 0.03) and steers whose sire was not in the reference (*p* = 0.04), but being purebred or having a reference sire did not affect r_sa_ (*p* > 0.10). Influence from minor *Bos taurus* breeds did not appear to affect CC or r_sa_, which were similar for steers composed of only Cycle VII breeds and those with some contribution from other *Bos taurus* breeds (*p* > 0.24). Steers sired by any other *Bos taurus* breed had a CC and r_sa_ similar to Angus-sired steers. Steers sired by all *Bos indicus*-influenced breeds had CC and r_sa_ lower than Angus-sired steers (*p* < 1e^−3^), but Brangus-sired steers had higher CC and r_sa_ than steers sired by the other *Bos indicus*-influenced breeds (*p* < 0.003). Sire breed differences were less for agreement with genotypes called from transcript sequence. Correlations between the imputed sequence and transcript genotype calls were not different for Angus, other *Bos taurus*, and Brangus-sired steers (*p* > 0.11). Correlations for Brahman-sired steers were less different from the Angus-sired steers (*p* = 0.04) than Beefmaster- (*p* = 0.002) or Santa Gertrudis-sired steers (*p* < 3e^−5^). Sire breed differences in correlations tested on a log scale (−log(1−r)), however, revealed some differences among *Bos taurus* breeds (Table 3) that were not evident when testing differences on the correlation scale.

### 3.3. Genomic Prediction

The three traits examined in this study, birth weight, postweaning gain, and marbling score, were all estimated to be at least moderately heritable. Heritability estimates were always greatest with pedigree relationships and the complete set of GPE phenotypes, followed by genomic relationships using the combined HD and F250 with phenotypes of genotyped GPE animals (Table 4). Functional content from the F250 explained more variation than the 50K marker set, but less than the full set of variants. Sets with a few hundred variants selected after permutation to eliminate variants with consistently large, spurious effects [37,38], explained approximately 2/3^rds^ the variation explained by the full variant set.

The pedigree and genomic BLUP with all variants included the sequenced steers’ data to predict (genomic) the estimated breeding values (G) (EBV). The steers’ phenotypes and genotypes were eliminated from analyses with variant subsets in order to compute variant effects that were not directly influenced by the steers’ data. Molecular breeding values from applying variant effects to steers’ genotypes had stronger correlations to their GEBV than to their pedigree EBV (Table 5). In all cases, correlations between MBV and (G) EBV were similar (within SE) using steers’ genotypes imputed with pedigree from their assayed genotypes or imputed from downsampled sequence. Correlations between MBV using either set of genotypes were > 0.96 (Table S4).

## 4. Discussion

Existing WGS available from steers produced by the multi-breed, industry-representative USMARC GPE project was downsampled to mimic low-pass sequencing, and provide an indication of how imputing low-pass sequence to the variants detected in a comprehensive haplotype reference panel might perform. For most of the steers sequenced, there was a strong agreement between genotypes imputed from downsampled sequence and genotypes called from SNP arrays and transcriptome sequence.

Five steers, however, had noticeably low agreement with the SNP array genotypes. This lack of agreement was initially indicated by genotype probabilities included in imputation results, which were summarized into a call confidence score for each individual. Extracting more complete records from the USMARC database revealed that four of the five low CC, low-agreement steers were twins to another calf. As the sequenced DNA was extracted from blood, the twins’ DNA would have included DNA from their co-twin, due to blood cell chimerism resulting from twins sharing blood across placental membranes [39,40]. The fifth low-confidence, low-agreement steer might have been a single-birth twin, whose co-twin embryo was lost early in pregnancy [39,40,41]. The CC score summarizing imputed genotype probabilities at least provides an indication of imputation accuracy, and possible issues with the sequenced DNA. Reasons for low CC scores included insufficient sequence reads to match reference haplotypes, missing reference haplotypes to match sequence reads, and contamination resulting in sequence matching conflicting reference haplotypes. As DNA extracted from twins’ blood is contaminated, low CC scores might indicate infertile single-birth heifers that were co-twins to a male embryo [41]. Further confirmation might be the presence of Y-chromosome sequence in DNA from the heifer’s blood [42], and higher CC with no Y sequence in the DNA extracted from other tissue.

Lower CC for *Bos indicus*-influenced steers suggests that haplotypes that match their sequence are missing from the reference panel. Although the reference panel contains more Brahman cattle than cattle from several *Bos taurus* breeds, PCA shows separation between Brahman that were influential in Australia and some Brahman sampled from the U.S. industry for GPE. Additionally, the Brahman and *Bos taurus* contributions to the Beefmaster (25% Hereford, 25% Shorthorn, 50% Brahman) and Santa Gertrudis (62.5% Shorthorn, 37.5% Brahman) breeds might be isolated. Both breeds descended from narrow bases, Beefmaster from a single closed herd that originated with Brahman bulls mated to Hereford and Shorthorn cows [43], and Santa Gertrudis from a single bull mated to F_1_ Brahman x Shorthorn heifers [44]. Both breeds allow grading up through mating Beefmaster or Santa Gertrudis bulls to undocumented females, but do not allow re-creating the composites from unrelated cattle representing the contributing breeds. Brangus policy, however, allows mating registered Angus (black and red) and Brahman to create the 62.5% Angus, 37.5% Brahman composite, which might maintain stronger connections to the contributing breeds, and explain Brangus as having somewhat higher CC and agreement between imputed genotypes and calls from SNP arrays and transcript sequence. Broader sampling of *Bos indicus*-influenced breeds for the imputation reference should increase the imputation accuracy for these cattle; further increases might be realized by reference construction and imputation strategies that consider the assembled genome of a Brahman cow [45].

The generally strong agreement between genotypes imputed from downsampled GPE steers and genotypes called from SNP arrays and transcript sequence certainly suggests imputation from low-pass sequence is a viable approach to genotyping sequence variants. Having a sequence of influential GPE animals in the haplotype reference, including sires of 20% of these steers, contributes to the quality of imputation. Further evaluation outside of GPE is needed to determine suitability of the current reference for imputing sequence genotypes of current seedstock and commercial crossbred cattle. Existing SNP array genotypes on current commercial and seedstock cattle might be useful to identify additional animals who would be informative in the haplotype reference panel. Genomic relationships among commercial calves, seedstock influencing those calves, and animals in the current reference could reveal influential seedstock lowly related to cattle in the current reference. Following [24,46,47,48], a more refined approach might infer haplotypes from array genotypes, prioritize the haplotypes based on frequency and existing coverage, then prioritize additions to the reference to add sequence to the highest frequency haplotypes that are lacking coverage.

The strong agreement between imputed and array genotypes allowed predicting steer MBV with imputed genotypes that agreed with MBV from variant effects, applied to those array genotypes. Even with the loss of assayed variants that were not imputed, correlations with pedigree EBV and GEBV using all assayed variants were similar for MBV computed with both array and imputed genotypes. Agreement was stronger with GEBV, predicted with available phenotypes for genotyped GPE animals, than with EBV, which used all available GPE phenotypes and pedigree records, but no genomic information. Agreement was similar for MBV that used either F250 or 50K genotypes, and was lower for small subsets of the F250. The small subsets selected, based on association with BW and PWG had a better agreement with corresponding (G) EBV than same-size randomly selected subsets, but agreement for MARB-associated and random subsets with MARB (G) EBV was similar. Previous work showed that small sets of SNP, selected with different approaches, might not fully explain variation within a population, but can predict across populations more accurately than larger sets of whole-genome SNP [1,2,3]. These subsets should be examined in cattle that are distant from the GPE population, before drawing conclusions about their effectiveness. Beyond this, including functional variants imputed from low-pass sequence that are not interrogated by the F250 might be considered.

The smaller panels were proposed for low-cost genotyping arrays. For a similar cost, the genotypes could be imputed from low-pass sequence, while avoiding complications of array design and development. Imputing the full set of variants detected in the haplotype reference from low-pass sequence is relatively straightforward and can capture individual variants within variant-dense regions where close, interfering SNP preclude designing probes for genotyping arrays. Especially important for low-frequency variants, imputed genotypes can be called from matches to haplotype reference sequence, without the need for sufficient data to train clustering algorithms to call array genotypes. Somewhat similar to selecting variants to probe with an array, a manageable number of variants might be selected from the full set of imputed genotypes for genomic analysis. Unlike an array, the set of variants extracted is flexible, without redesigning and manufacturing a different array.

Genotypes for the 50K variants imputed from low-pass sequence could be extracted to include with existing array genotypes for genome-enhanced national cattle evaluation (NCE). National cattle evaluation might be extended to traits that are not routinely recorded, and cattle that are not usually evaluated if the LD-dependent 50K were replaced with causal variants. Current within-breed NCE rely on consistent LD between 50K and unknown causal variants for genomic predictions of routinely recorded traits in seedstock cattle. Causal variants, at least functional variants that are likely to affect phenotype, could reduce reliance on LD and enable genomic predictions that are more robust across populations [1,2,3]. This could allow genomic prediction of difficult-to-measure traits, based on records from intensely measured herds, and predictions for commercial cattle that are not included in seedstock evaluations. Reliable predictions to guide sorting commercial cattle for management and marketing could help to justify the expense of low-pass sequencing. Phenotypes and genotypes imputed from low-pass sequence on commercial cattle could further increase reliability of genomic prediction for both commercial and seedstock cattle, if data-sharing mechanisms are in place to allow commercial records to inform NCE. Similarly, reducing per-sample costs of low-pass sequencing to a point well under current array costs, perhaps through less expensive DNA extraction and sequencing library preparation, might encourage more complete genotyping of seedstock and commercial calves, and provide even more data to support accurate genomic prediction.

## 5. Conclusions

Existing genome sequence from individuals that also had transcriptome sequence and SNP array genotypes provided an opportunity to assess low-pass sequence and imputation to sequence variants. Downsampling mimicked low-pass sequencing, and genotypes for nearly 60 million variants detected in a broad haplotype reference panel were imputed. Agreement between imputed genotypes and genotypes called from the SNP arrays and transcriptome sequence was generally strong, somewhat stronger for *Bos taurus* than *Bos indicus*-influenced cattle. Expanding the reference panel to include more *Bos indicus*-influenced haplotypes might increase agreement for those cattle. Further evaluation of relationships among current industry cattle and individuals in the reference panel might reveal additional cattle that might contribute to the reference. Owing to the agreement between SNP array and imputed genotypes, MBV with array variant effects applied to either array or imputed genotypes were similar. Molecular breeding values that more completely explained sequence variation that affect phenotypic variation might be obtained by transitioning genomic prediction from the limited set of variants interrogated by SNP arrays, to functional variants detected in sequence. These variants could currently be imputed from low-pass sequence at a cost similar to the least expensive SNP arrays. Further developments that could lower costs of obtaining low-pass sequence and increase accuracy of imputation and genomic prediction might make genotyping from low-pass sequence more accessible and worthwhile for seedstock and commercial cattle.

## Figures and Tables

**Table 1:** Animals genotyped in the Germplasm Evaluation Project.

SNP Array	N
BovineSNP50 ^a^	9930
BovineHD ^b^	1547
GGP ^c^ -F250	2339
GGP-50K	3068
GGP ^d^	5083

^a^ BovineSNP50 (Illumina, Inc.) versions 1 and 2; ~54,000 SNP. ^b^ BovineHD (Illumina, Inc.); ~780,000 SNP. ^c^ GeneSeek Genomic Profiler (GGP) F250 (Neogen, Inc.); ~220,000 putative functional SNP. ^d^ GGP versions 1 to 4; ~20,000 to 75,000 SNP.

**Table 2:** Functional classification of variants detected in the cattle haplotype reference panel.

	Reference ^b^		SNP Array ^c^	
Classification ^a^	Variants	Genes	Variants	Genes
Protein-changing	332,714	21,066	29,519	10,673
High impact	14,773	9084	545	509
Non-synonymous SNP	318,269	20,978	29,011	10,576
Potentially regulatory	327,357	18,110	13,072	8076
Untranslated region (UTR)	318,495	15,288	12,447	7557
Non-coding RNA	8940	2822	627	519
Intergenic	38,694,029		396,306	
Intronic	19,533,912		272,510	
Total	59,198,026	21,334	715,402	10,683

^a^ Variants classified with snpEff v4.3 using ensembl ARS-UCD1.2.96 annotation. ^b^ Variants detected in the cattle haplotype reference panel and imputed from the low-pass sequence. ^c^ Autosomal and pseudo-autosomal variants detected in the reference panel and with usable SNP array genotypes in Germplasm Evaluation Project cattle.

**Table 3:** Sire-breed differences among correlations between genotypes imputed from downsampled sequence and called from transcript sequence.

	Correlation (r) Scale			−log(1−r) Scale		
Sire Breed	Effect ^a^	SE	*p* Value	Effect ^a^	SE	*p* Value
Red Angus	4.60 × 10^−4^	4.92 × 10^−3^	9.26 × 10^−1^	−0.07	0.21	7.52 × 10^−1^
Brahman	−2.79 × 10^−2^	3.81 × 10^−3^	1.59 × 10^−9^	1.86	0.16	8.17 × 10^−16^
Beefmaster	−2.08 × 10^−2^	3.36 × 10^−3^	1.01 × 10^−7^	1.60	0.14	1.78 × 10^−15^
Brangus	−1.05 × 10^−2^	3.11 × 10^−3^	1.37 × 10^−3^	1.15	0.13	1.25 × 10^−11^
Charolais	−2.02 × 10^−3^	3.36 × 10^−3^	5.50 × 10^−1^	0.36	0.14	1.55 × 10^−2^
ChiAngus	−2.48 × 10^−3^	4.92 × 10^−3^	6.16 × 10^−1^	0.40	0.21	6.38 × 10^−2^
South Devon	−2.54 × 10^−3^	6.60 × 10^−3^	7.02 × 10^−1^	0.44	0.28	1.22 × 10^−1^
Gelbvieh	−1.63 × 10^−3^	3.81 × 10^−3^	6.71 × 10^−1^	0.29	0.16	7.83 × 10^−2^
Hereford	−6.70 × 10^−4^	3.55 × 10^−3^	8.51 × 10^−1^	0.13	0.15	4.10 × 10^−1^
Limousin	−1.80 × 10^−3^	6.60 × 10^−3^	7.86 × 10^−1^	0.34	0.28	2.34 × 10^−1^
Maine-Anjou	−3.20 × 10^−4^	4.92 × 10^−3^	9.48 × 10^−1^	0.09	0.21	6.72 × 10^−1^
Salers	−2.75 × 10^−3^	3.81 × 10^−3^	4.74 × 10^−1^	0.47	0.16	5.95 × 10^−3^
Braunveih	−3.89 × 10^−3^	4.92 × 10^−3^	4.33 × 10^−1^	0.61	0.21	5.66 × 10^−3^
Simmental	−2.57 × 10^−4^	4.21 × 10^−3^	9.52 × 10^−1^	0.07	0.18	6.79 × 10^−1^
Shorthorn	−1.25 × 10^−3^	3.55 × 10^−3^	7.62 × 10^−1^	0.24	0.15	1.22 × 10^−1^
Santa Gertrudis	−2.21 × 10^−2^	3.36 × 10^−3^	2.34 × 10^−8^	1.66	0.14	5.55 × 10^−15^

^a^ Difference from Angus.

**Table 4:** Restricted maximum likelihood heritability (h^2^) estimates for birth weight, postweaning gain, and marbling score using pedigree and different genomic relationship matrices.

	Birth Weight		Postweaning Gain		Marbling Score	
Relationship ^a^	h^2^ (SE)	N	h^2^	*n*	h^2^	*n*
Pedigree ^a^	0.595 (0.008)	78,625	0.526 (0.010)	68,846	0.538 (0.018)	33,850
G_all_^b^	0.573 (0.011)	16,512	0.474 (0.013)	16,144	0.508 (0.017)	10,898
G_F250_^c^	0.545 (0.011)	16,440	0.442 (0.012)	16,068	0.471 (0.016)	10,822
G_F250s_^d^	0.380 (0.023)	16,440	0.270 (0.019)	16,068	0.342 (0.021)	10,822
G_F250r_^e^	0.066 (0.007)	16,440	0.062 (0.007)	16,068	0.105 (0.009)	10,822
G_50K_^f^	0.519 (0.011)	16,440	0.437 (0.012)	16,068	0.466 (0.016)	10,822

^a^ Pedigree BLUP including downsampled steers. ^b^ 748,804 autosomal and pseudo-autosomal variants from GGP-F250 and BovineHD arrays, filtered for >0.95 call rate and pedigree imputation accuracy. Genomic BLUP (GBLUP) included downsampled steers. ^c^ 116,472 filtered variants from GGP-F250. GBLUP excluded downsampled steers. ^d^ GGP-F250 subsets selected for trait-specific effects: 551 birth weight; 585 postweaning gain; and 698 marbling score. GBLUP excluded downsampled steers. ^e^ Randomly selected GGP-F250 subsets, same size as trait-specific subsets. GBLUP excluded downsampled steers. ^f^ 51,496 BovineHD variants common with BovineSNP50 array. GBLUP excluded downsampled steers.

**Table 5:** Correlations (SE) between molecular breeding values (∑ (marker effect estimates × genotypes)) and predicted breeding values.

	Birth Weight		Postweaning Gain		Marbling Score	
Predictions Using Imputed SNP Array Genotypes						
	**Pedigree ^a^**	**G_all_^b^**	**Pedigree**	**G_all_**	**Pedigree**	**G_all_**
G_F250_^c^	0.738 (0.061)	0.904 (0.037)	0.779 (0.055)	0.881 (0.041)	0.770 (0.057)	0.926 (0.032)
G_F250s_^d^	0.555 (0.079)	0.681 (0.067)	0.653 (0.069)	0.714 (0.063)	0.655 (0.069)	0.750 (0.059)
G_F250r_^e^	0.379 (0.090)	0.481 (0.083)	0.344 (0.093)	0.385 (0.090)	0.629 (0.070)	0.741 (0.058)
G_50K_^f^	0.710 (0.063)	0.888 (0.039)	0.785 (0.055)	0.886 (0.040)	0.794 (0.053)	0.950 (0.026)
Predictions Using Genotypes Imputed from Low-Coverage Sequence						
G_F250_^c^	0.680 (0.067)	0.866 (0.044)	0.779 (0.055)	0.887 (0.040)	0.769 (0.057)	0.936 (0.030)
G_f250s_^d^	0.531 (0.081)	0.634 (0.073)	0.635 (0.071)	0.722 (0.063)	0.641 (0.071)	0.738 (0.062)
G_F250r_^e^	0.286 (0.100)	0.390 (0.094)	0.332 (0.096)	0.395 (0.094)	0.649 (0.071)	0.776 (0.057)
G_50K_^f^	0.676 (0.067)	0.866 (0.044)	0.805 (0.052)	0.903 (0.037)	0.760 (0.058)	0.941 (0.029)

^a^ Breeding values predicted by BLUP with pedigree relationships, including downsampled steers. ^b^ Breeding values predicted by BLUP with genomic relationships computed using genotypes of 748,804 autosomal and pseudo-autosomal variants from the GGP-F250 and BovineHD arrays, including downsampled steers. ^c^ Molecular breeding values of the downsampled steers predicted with effects of 116,472 GGP-F250 variants solved from GBLUP, excluding downsampled steer records. ^d^ GGP-F250 subsets selected for trait-specific effects: 551 birth weight; 585 postweaning gain; and 698 marbling score. GBLUP excluded downsampled steers. ^e^ Randomly selected GGP-F250 subsets, same size as trait-specific subsets. GBLUP excluded downsampled steers. ^f^ 51,496 BovineHD variants common with BovineSNP50 array. GBLUP excluded downsampled steers.

## Supplementary Materials

The following are available online at https://doi.org/10.5281/zenodo.4147100. Table S1: Sup1.tsv, tab separated text list of animals in imputation reference, containing ID, SRR Accessions, Source, Breed, and number of downsampled progeny examined in this study; Table S2: Sup2.vcf, VCF-formatted text containing snpEff annotation of variants imputed from low-pass sequence; Table S3: Sup3.tsv, tab separated text summarizing number of variants, low-confidence variants, and variant spacing in 1 Mb intervals; Figure S1: steerworkflow.pdf, diagram depicting processes to obtain imputed genotypes, (G) EBV and MBV of the study steers; Table S4; Sup4.tsv, tab separated text containing correlations between array- and sequence-based MBV. The loimpute software is available from https://gitlab.com/gencove/loimpute-public.
